# Super‐resolved highly multiplexed immunofluorescence imaging for precise protein localization and podocyte ultrastructure

**DOI:** 10.1111/jcmm.70066

**Published:** 2024-09-27

**Authors:** Florian Siegerist, Svenja Kitzel, Nihal Telli, Juan Saydou Dikou, Vedran Drenić, Christos E. Chadjichristos, Christos Chatziantoniou, Nicole Endlich

**Affiliations:** ^1^ Department of Anatomy and Cell Biology University Medicine Greifswald Greifswald Germany; ^2^ Department for Pediatrics University Medicine Greifswald Greifswald Germany; ^3^ Center of High‐End Imaging NIPOKA GmbH Greifswald Germany; ^4^ UnitéMixte de Recherche (UMR)‐S1155, National Institute for Health and Medical Research (INSERM), Tenon Hospital Sorbonne Universités Paris France

**Keywords:** kidney disease, multiplex immunofluorescence, super resolution microscopy

## Abstract

Deep insights into the complex cellular and molecular changes occurring during (patho‐)physiological conditions are essential for understanding the interactions and regulation of proteins. This understanding is crucial for research and diagnostics. However, the effectiveness of conventional immunofluorescence and light microscope, tools for visualizing the spatial distribution of cells or proteins, are limited both in resolution and multiplexity in complex tissues. This is mainly due to challenges such as the spectral overlap of fluorophore wavelengths, a limited range of antibody types, the inherent variability of samples and the optical resolution limit. The herein demonstrated combination of multiplex immunofluorescence imaging and super resolution microscopy offers a solution to these limitations by enabling the identification of different cell types and precise subcellular localization of proteins in tissue sections. In this study, we demonstrate the cyclic staining and de‐staining of paraffin kidney sections, making it suitable for routine use and compatible with super‐resolution microscopy for podocyte ultrastructural studies. We have further developed a computerized workflow for data processing which is accessible through available reagents and open‐access code. As a proof of principle, we identified CDH2 as a marker for cellular lesions of sclerotic glomeruli in the nephrotoxic serum nephritis mouse model and cross‐validated this finding with a human Nephroseq dataset indicating its translatability. In summary, our work represents an advance in multiplex imaging, which is crucial for understanding the localization of numerous proteins in a single FFPE kidney section and the compatibility with super‐resolution microscopy to study ultrastructural changes of podocytes.

## 1 INTRODUCTION

**FIGURE 1:**
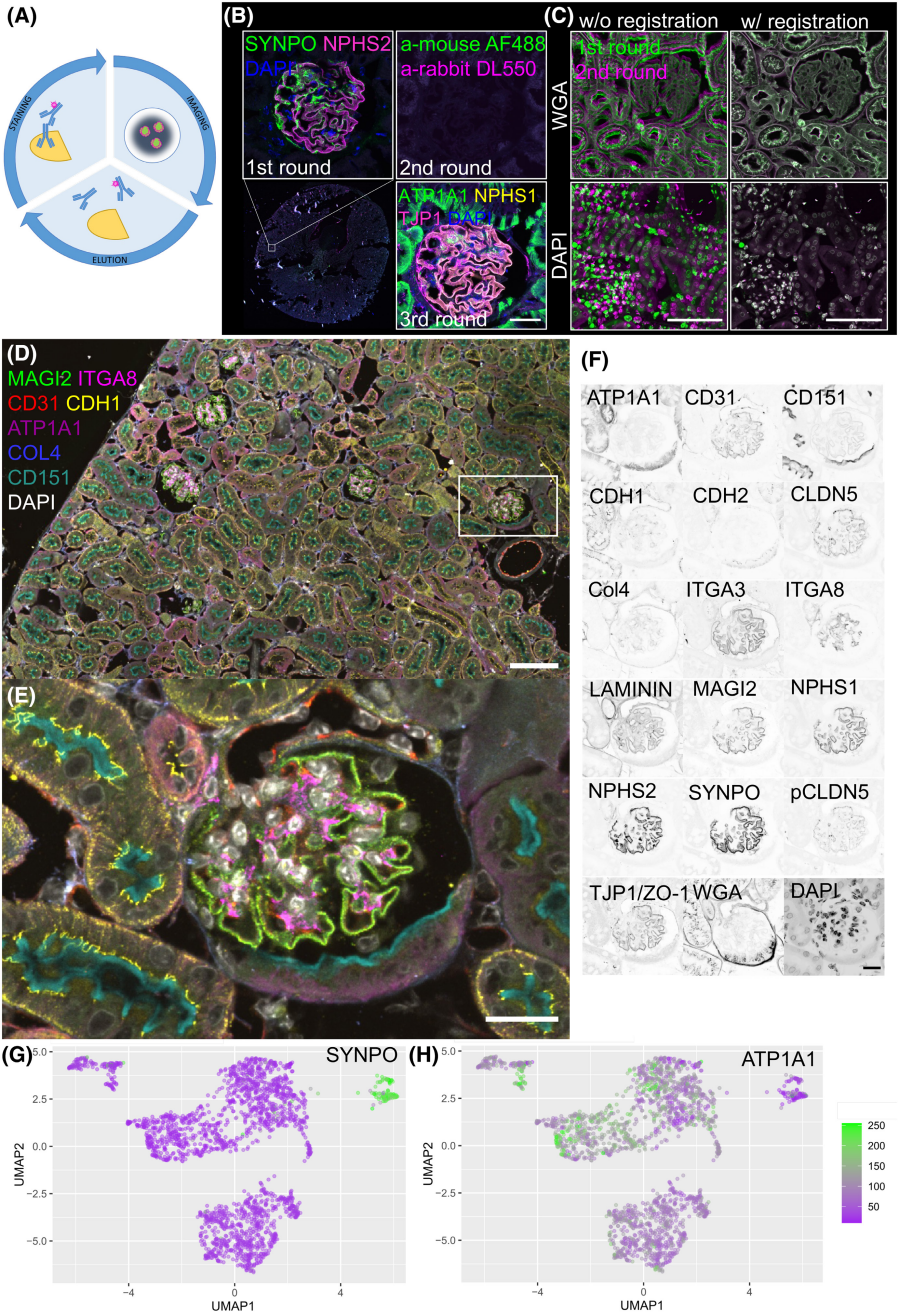
The scheme in (A) shows the general approach. A secondary immunofluorescence is performed, antibody binding is visualized with fluorescence microscopy, and the antibodies finally eluted from the section therefore enabling a new antibody binding and imaging cycle. The panel in (B) demonstrates the full elution of antibodies from a tissue section. A mouse kidney section was incubated with anti‐synaptopodin (SYNPO) and anti‐podocin (NPHS2) primary antibodies, which were detected with anti‐mouse and anti‐rabbit secondary antibodies (1st round). After eluting the first secondary immunofluorescence (IF), the section was incubated with the secondary antibodies only, which showed no binding of the secondary antibodies (2nd round). Finally, the same section was again incubated with primary and secondary antibodies which demonstrated the integrity of the antigen on the section (3rd round). The images in (C) show the functionality of the image registration algorithm to precisely align the consecutive imaging rounds. (D–F) show a merged mIF dataset for 17 different proteins, and DAPI as a nuclear marker of a mouse kidney section. Cells were segmented and single‐cell protein mean fluorescence intensities were retrieved. A UMAP was calculated from the resulting dataset, demonstrating precise clustering according to diverse cell‐identities as exemplarily shown for the upper right podocyte cluster SYNPO as a marker protein (G); or tubular cells ATP1A1 (H). Scale bars represent 50 μm in B and C, and 20 μm in (D–F).

The cellular heterogeneity of the kidney presents challenges when analysing gene and protein expression. Although bulk omics can provide valuable insights, they have limitations in capturing the complete range of cell types involved in kidney (patho‐)physiology. While still expensive and therefore inaccessible to a lot of researchers, single‐cell RNA sequencing and spatial transcriptomics have emerged as powerful tools that enable the identification of individual cell types and their gene expression profiles in the kidney.^1^, ^2^ Immunofluorescence staining (IF) enables the sensitive localization of specific proteins in tissue sections. Besides this, IF has been used for gross glomerular morphometry^3^ or super‐resolved ultra‐morphometry of the filtration barrier.^4^ Traditional IF is limited by spectral overlap of the fluorophores and the limited diversity of antibody species, which restricts the number of channels that can be imaged on one section. This limitation has been addressed by the highly multiplexed immunofluorescence (mIF) methods like the one developed by Gut and colleagues (4i), which uses cyclic staining and de‐staining allowing the imaging of dozens of different proteins in cultured cells or tissue sections.^5^, ^6^ In the present study, we labelled proteins of interest with primary and secondary antibodies, which after being imaging with either confocal laser scanning microscopy or super‐resolution three‐dimensional (3D) structured illumination microscopy (3D‐SIM) are eluted from the sample (Figure 1A). This way, the sole factor that limits the number of imaging cycles is the number of working antibodies available. To make this method as accessible as possible for a broad spectrum of researchers, all reagents used are commercially available, the image analysis codes are published open‐source, and the imaging chambers are custom‐designed and 3D‐printed. Therefore, our approach opens new perspectives for inexpensive, rapid and more efficient evaluation of histological samples not only in research, but also in clinical diagnosis because a limited amount of tissue can be used for multiple analysis.

## 2 SHORT METHODS

### 2.1 Tissue processing and immunostaining

Mouse kidney tissue was fixed with 4% paraformaldehyde (PFA) for a previous study has been used as described before.^7^ Control rat tissue was fixed by retrograde aortic perfusion fixation with 4% PFA in deep anaesthesia (ketamine/xylazine). This animal experiment has been approved and was overseen by the LALLF Rostock, Germany (file number 7221.3‐1‐037/22‐1). After immersion fixation in 4% PFA overnight at room temperature (RT), the tissue was embedded in paraffin and 2 μm sections were collected on 22 × 22 mm #1.5 high‐precision coverslips coated with 2% (3‐Aminopropyl) triethoxysilane (Sigma‐Aldrich, Cat. 440140). After deparaffinization, heat‐induced epitope retrieval was performed by 5 min boiling in a pressure cooker in TRIS‐EDTA buffer pH 9. Coverslips were immobilized in custom‐designed and 3D‐printed coverslip holders (.stl files available at http://www.github.org/Siegerist). Sections were blocked with 1% BSA, 200 mM NH_4_Cl and 150 mM maleimide. Primary antibodies (diluted in 1% BSA; 200 mM NH_4_Cl) were incubated for 2 h at RT. Sections were washed with PBS, and secondary antibodies were incubated for 1 h at RT. AlexaFluor 555 or CF770‐conjugated wheat germ agglutinin (WGA) was added to the secondary antibody solution to a final concentration of 2 μg/mL. After several washes in PBS, cell nuclei were stained with 0.1 mg/mL DAPI. The sections were imaged in 700 mM N‐acetyl‐cysteine in H_2_O pH 7.4 on a FluoView3000 confocal laser scanning system (Olympus) equipped with a 20× 0.8 NA dry objective (UPLXAPO20X) or a UPLSAPO60XW NA 1.2 water immersion objective (validation experiments). After imaging, antibodies were eluted with 0.5 M L‐glycine, 3 M urea, 3 M GC; 70 mM TCEP in ddH_2_O (pH 2.5), blocked and re‐stained as described above.

Imaging with a 3D super‐resolution microscopy was performed by using a Nikon N‐SIM E system (Nikon Instruments, Japan) at NIPOKA GmbH (Greifswald, Germany). For overview images the 20×/0.75NA objective was used and the high‐resolution images were acquired by using 100×/1.35NA silicon immersion objective. The Nikon N‐SIM E system is equipped with 488,561 and 640 nm laser lines.

### 2.2 Quantitative image analysis

A script was established in the IJ1 language that registers and corrects the drift which can be found at http://www.github.org/Siegerist. Briefly, minima and maxima are detected with sub‐pixel accuracy in both respective images and translated to one another using a descriptor‐based registration algorithm.^8^ This process was iteratively repeated until all images were merged. From the overviews glomeruli were detected with a pretrained UNet, and individual cell nuclei with surrounding cytoplasm were segmented with a custom‐trained Deep Learning network (StarDist in QuPath v.0.4.3)^9^ as shown in Figure S9. Uniform Manifold Approximation and Projections (UMAPs) were generated in R‐Studio with the UMAP package.

## 3 RESULTS

### 3.1 Optimization of the 4i protocol for formalin‐fixed paraffin‐embedded (FFPE) kidney tissue

For cyclic staining, imaging and de‐staining (Figure 1A) of kidney, we improved tissue adhesion by coating coverslips with 3‐aminopropyltriethoxysilane which was superior to poly‐l‐lysine or uncoated slides (Figure S1). During elution, both secondary and primary antibodies were efficiently removed from their epitopes as shown in Figure 1B.

As exemplarily shown for mouse and human kidney tissue in Figures S2 and S3, in which the same glomerulus was imaged over three cycles, the staining and de‐staining worked efficiently without bleed‐through from previous rounds and the tissue morphology was unimpaired.

To establish an mIF antibody panel, we screened 44 antibodies on human and mouse kidney tissue in single immunostainings. We found 20 antibodies for mouse kidney tissue including tubular markers (ATP1A1, E‐cadherin, N‐cadherin, ezrin), glomerular markers (podocin, synaptopodin, nephrin, MAGI2, ITGA3, DACH1, CD151), tight junction proteins (TJP1, CLDN5, phospho‐CLDN5), endothelial markers (CD31) mesangial markers (ITGA8) and markers for extra cellular matrix (ECM) components (laminin, collagen IV) (Figure S4).

To reduce drift, staining and imaging cycles were performed in a custom 3D‐printed chamber. As drift could not be mechanically abrogated completely (Figure 1C), a marker that is consistently stained in every round is required to detect the shift. Since DAPI staining quality was inconsistent, independent on the number of staining cycles (Figure S5), we stained the surface using near‐infrared CF770‐conjugated wheat‐germ agglutinin which could be efficiently eluted and re‐stained consistently on the same section (Figure S6).

2D‐descriptor‐based image registration within the open‐source ImageJ ecosystem was used to precisely align the datasets of two subsequent imaging rounds in both DAPI and WGA channels (Figure 1C, overviews in Figure S7).

Large tile scans of tissue sections were acquired on a confocal laser scanning system and stitched to high‐resolution overviews. To retrieve single‐cell fluorescence data for all antigens, we established a workflow that preprocesses the raw images to generate precisely aligned multichannel data (Figure S8A).

Subsequently, the section was virtually dissected into glomeruli with a custom‐trained U‐Net and in single cells with a StarDist network (Figure S8B). For every cell segmented on the slide, morphometric measures (nuclear size, roundness and nuclear‐to‐cytoplasmic ratio), classification to a tissue compartment (glomerulus vs. tubulointerstitium) and fluorescence intensity quantifications were reported. In this way, single‐cell co‐expression analysis from a few thousand cells was enabled, as demonstrated for NPHS2, SYNPO, ATP1A1 and TJP1 with NPHS1, respectively (Figure S8C). For a typical overview of a tissue section of 700×700 μm as shown in Figure 1D, we could retrieve single‐cell data from at least 3000 cells.

As shown in the magnified view in Figure 1E, the resolution and orientation of the cyclically acquired images was found to be sufficient to capture the details of the subcellular localization of the proteins studied. Notable examples are the distinct localization of CD151 at the brush border, ATP1A1 to the basolateral cell membrane and collagen IV to the basement membrane. All three glomerular cell types could be identified by their staining such as MAGI2 (podocytes), ITGA8 (mesangial cells) and CD31 (glomerular endothelial cells), respectively. Figure 1F shows the single‐channel overview of all targets stained on this tissue section. The image analysis workflow described above generated a highly multidimensional dataset that was used to cluster cells in an unbiased manner according to their expression patterns and morphometric measures using UMAP. As shown in Figure 1G,H, four well‐separated clusters could be identified. The smallest cluster in the upper right could be identified as the podocyte cluster by the synaptopodin expression (SYNPO, Figure 1G). The larger cell clusters represented tubular cells (ATP1A1, Figure 1H).

### 3.2 Applying mIF in a crescentic glomerulonephritis model identified CDH2 as a marker for cellular glomerular crescents

**FIGURE 2:**
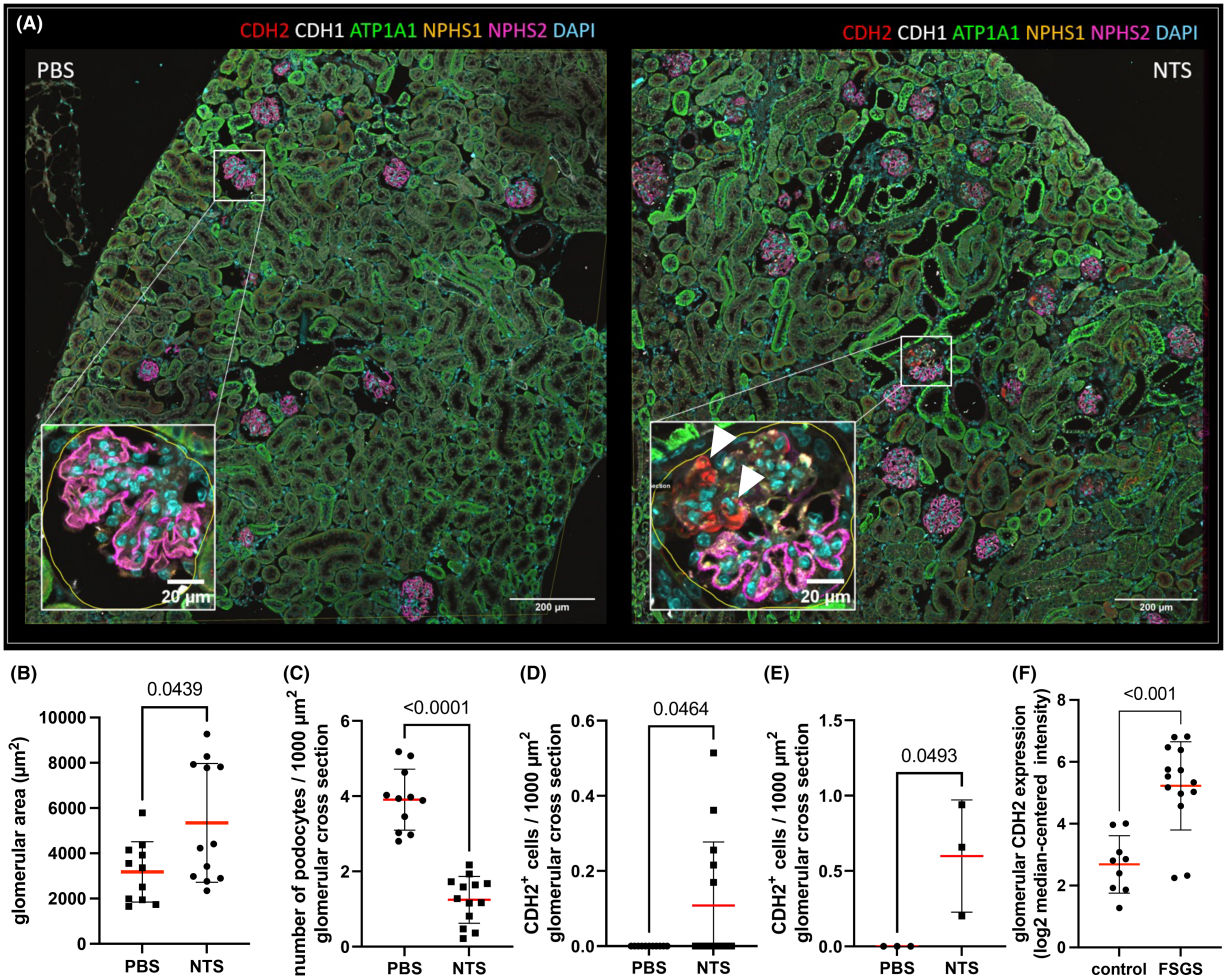
(A) shows a comparison of a control mouse section (PBS) with an NTS‐injected mouse. We demonstrate glomerular hypertrophy as shown by increase of the mean glomerular cross‐sectional area (B). Additionally, decreased podocyte density in the NTS‐injected animals (C) was demonstrated indicating podocyte depletion. Interestingly, we found an increase of CDH2‐expressing glomerular cells only in NTS‐injected animals (arrowheads in A), which could be quantified as shown in (D). To verify this finding, the analysis was extended to three animals per group in which the appearance of CDH2+ cells in the glomerular tuft was consistent (E). Within the Nephroseq database, CDH2 was enriched in glomeruli of patients diagnosed for FSGS (F).

To benchmark our workflow, we used the antibody panel in murine nephrotoxic serum nephritis (NTS), a model for crescentic glomerulonephritis. The kidney of the NTS‐injected animal showed glomerular hypertrophy (Figure 2B) as well as a loss of podocytes (depletion of triple NPHS1‐, NPHS2‐ and MAGI2‐positive intraglomerular cells). In glomerular cross‐sections, we quantified 1.2 podocytes/1000 μm^2^ in NTS animals versus 3.9 podocytes/1000 μm^2^ in control animals (*p* < 0.0001; Figure 2C).  The proximal tubule marker CDH2 (*N*‐cadherin) was found to be expressed particularly in cellular lesions of sclerotic glomeruli (0.6 CDH2+ cells/1000 μm)^2^ in the NTS tissue (*p* = 0.0464) versus no positive cell was found in the control animal (Figure 2D). This finding was verified with standard immunofluorescence for CDH2 on an extended cohort of six animals (Figure 2E). In isolated glomeruli of FSGS patients, 5.4‐fold upregulation of the CDH2 mRNA in contrast to healthy kidneys was seen (Figure 2F, Hodgin FSGS Glom dataset obtained from the Nephroseq database).

### 3.3 Super‐resolved mIF uncovers glomerular filtration barrier details

**FIGURE 3:**
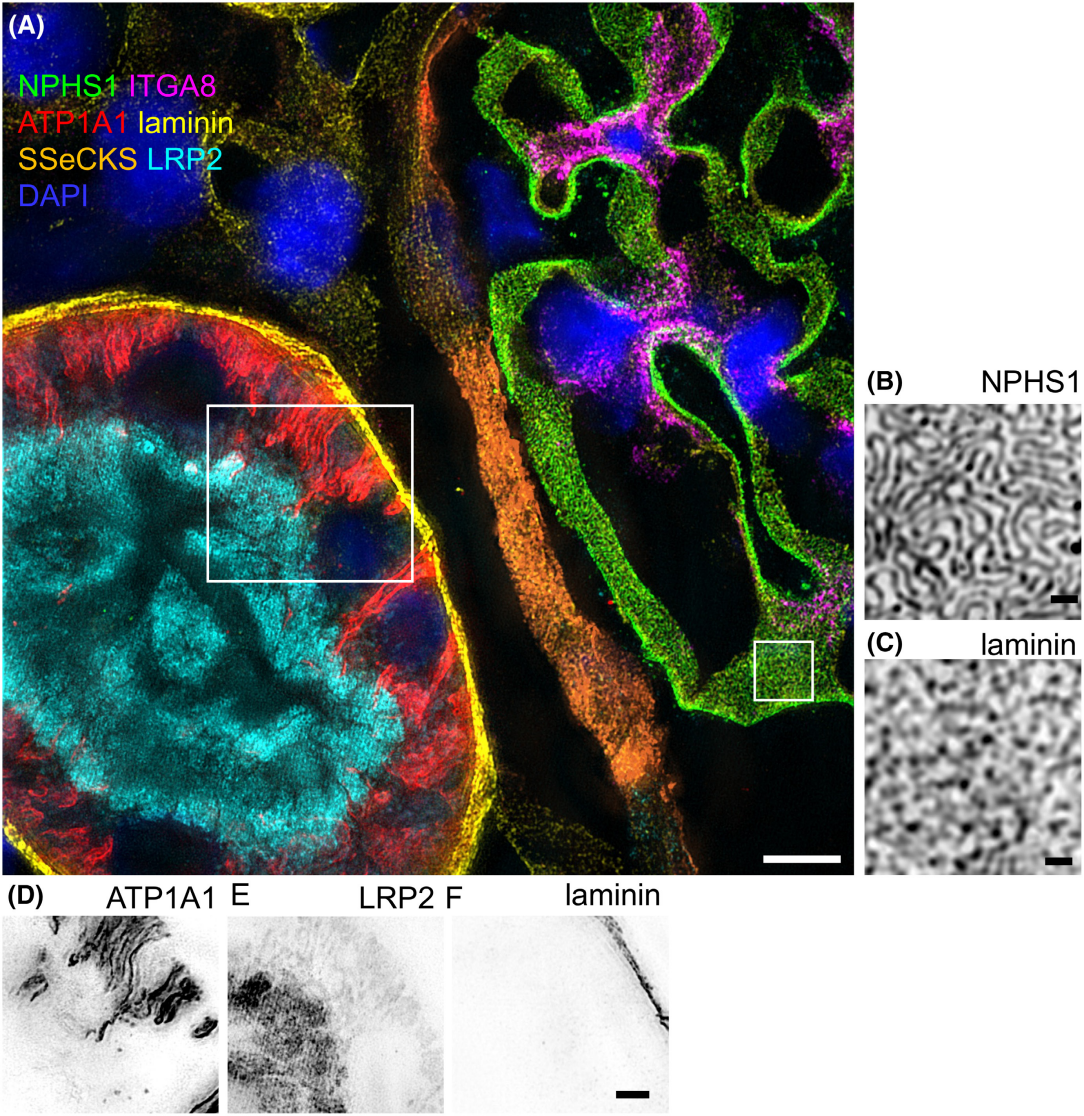
The image in (A) shows a super‐resolved mIF dataset consisting of six different tubular and glomerular markers. Magnifications in (B) show that the resolution was high enough to determine podocyte foot processes on the laminin positive GBM (C). Besides glomerular proteins, also tubular markers like ATP1A1 in the basolateral membrane (D) and LRP2 in the apical cell membrane (E), as well as laminin in the tubular basement membrane (F) could be precisely resolved from one another. Scale bars represent 5 μm in A, 500 nm in B and C and 2 μm in (D–F).

To reveal the subtle morphological details of the filtration barrier, in particular the podocyte foot processes and meandering filtration slits between them, we analysed rat kidney sections using 3D‐SIM. The tissue preservation allowed resolution of the filtration slit by staining for podocin, even after multiple previous staining, imaging and elution cycles. As shown in the overview in Figure 3A, we could super‐resolve both glomerular (nephrin, integrin alpha 8 and SSeCKS) and tubular marker proteins (ATP1A1 and LRP2), as well as ubiquitously expressed ECM proteins (laminin). As demonstrated in the magnified images in Figure 3B,C, the resolution was high enough to distinguish individual podocyte foot processes, therefore enabling podocyte foot process morphometry. These results demonstrate the direct integration of super resolution microscopy into the mIF workflow.

## 4 DISCUSSION

Direct single‐cell correlation of multiple proteins of interest is typically limited in 3‐4‐plex classic IF due to the problem of a limited number of different species for antibody production. However multiplex immunofluorescence analysis has the potential to significantly increase the amount of information on protein expression, localization as well as in relation to other proteins in single kidney section and therefore give a more comprehensive view of the tissue microenvironment. Until today, there are different techniques published which allow a multiplex staining of cells and tissue. Of the techniques available, the one presented by Gut et al. works with an adaption of routine secondary immunofluorescence utilizing commercially available antibodies and fluorophores.^6^ Within the kidney field, also other mIF approaches have been published, like the one presented by Rajagopalan et al. However, this specific technique requires the direct conjugation of antibodies with modified nucleic acids, therefore increasing hands‐on time, complexity and costs of an mIF experiment.^5^

Herein, our goal was to develop a method with the smallest barriers possible to retrieve highly mIF information from kidney samples that are prepared using standard protocols. Therefore, we used only commercially available antibodies, so that the protocol presented here can be easily reproduced.

In this study, we furthered our long‐term objective of maximizing the comprehensive acquisition of data from individual tissue sections. Building upon the scoMorphoFISH methodology, which integrated single‐cell, single‐mRNA quantification with deep learning‐enabled and super‐resolved morphometry,^9^ we have augmented this approach by significantly enhancing the information retrieval. This augmentation is achieved through the incorporation of multiple protein localization with mIF, protein abundance and with super‐resolved morphological information, thus enriching the breadth and depth of analytical insights obtained from tissue samples.

We and others have extensively shown that 3D‐SIM is a method that is suitable to retrieve ultrastructural information from routinely processed kidney samples both from patient samples and from animal models.^4^, ^9^, ^10^, ^11^, ^12^, ^13^ In principle, this methodology should be applicable to any super‐resolution technique compatible with FFPE tissue sections. While various super‐resolution methods, such as direct stochastic optical reconstruction microscopy and stimulated emission depletion microscopy, are compatible with FFPE samples, 3D‐SIM requires comparatively minimal adjustment of the standard workflow.^14^, ^15^, ^16^ Subsequent investigations will be necessary to determine the compatibility of more recent advancements, such as expansion‐enhanced super‐resolution‐enabled radial fluctuation nanoscopy,^17^, ^18^, ^19^ with the relatively harsh antibody elution procedure utilized in this study.

Besides the establishment of the protocol for FFPE sections, we found CDH2 as a marker upregulated in cellular crescents of murine NTS‐nephritis. While in healthy animals, CDH2 is localized in the apical junctional complex of proximal tubule cells, its distribution changed in glomeruli of NTS‐injected animals. Similarly it has been described by other groups and us, that cells at the interface between parietal epithelial and proximal tubular cells can be activated and recruited to injured glomeruli so that tubular markers are de novo expressed in injured glomeruli.^20^, ^21^ Interestingly, this finding was consistent with a microarray dataset of laser‐captured glomeruli from archived FSGS patients.^22^ Therefore, following studies will focus on the establishment of CDH2 as a marker protein for the classification of glomerular injury in patients.

One important advantage of our established method is that all reagents are commercially available and commonly used in classic IF, making it easy to extend existing workflows. Taken together, we present a straightforward addition to massively expand the information obtained from a single FFPE section with reagents available in most laboratories already working with IF. Therefore, this workflow is a valuable addition to cost‐efficiently increase the information retrieved from single tissue sections.

## AUTHOR CONTRIBUTIONS

**Florian Siegerist:** Conceptualization (equal); formal analysis (equal); investigation (equal); methodology (equal); project administration (equal); software (equal); visualization (lead); writing – original draft (lead). **Svenja Kitzel:** Investigation (equal); methodology (equal); visualization (supporting); writing – original draft (supporting). **Nihal Telli:** Investigation (supporting); visualization (supporting). **Juan Saydou Dikou:** Investigation (supporting); methodology (supporting); software (equal). **Vedran Drenić:** Investigation (supporting); visualization (supporting). **Christos E. Chadjichristos:** Investigation (supporting). **Christos Chatziantoniou:** Investigation (supporting); project administration (supporting). **Nicole Endlich:** Conceptualization (equal); funding acquisition (lead); project administration (equal).

## CONFLICT OF INTEREST STATEMENT

N.E. serves as CEO, V.D. is an employee and F.S. hold shares of NIPOKA GmbH, a company commercializing the PEMP approach.

## Supporting information


Data S1.

## Data Availability

The data will be made fully available upon reasonable request.
